# Treatment with the copper compound CuATSM has no significant effect on motor neuronal pathology in patients with ALS

**DOI:** 10.1111/nan.12919

**Published:** 2023-07-04

**Authors:** Yue Yang, Dominic Rowe, Heather McCann, Claire E. Shepherd, Jillian J. Kril, Matthew C. Kiernan, Glenda M. Halliday, Rachel H. Tan

**Affiliations:** ^1^ Brain and Mind Centre University of Sydney Sydney New South Wales Australia; ^2^ Faculty of Medicine and Health, School of Medical Sciences University of Sydney Camperdown New South Wales Australia; ^3^ Institute of Clinical Neurosciences Royal Prince Alfred Hospital Sydney New South Wales Australia; ^4^ Macquarie University Centre for Motor Neuron Disease Research, Faculty of Medicine, Health and Human Sciences Macquarie University Sydney New South Wales Australia; ^5^ Dementia Research Centre, Macquarie Medical School Macquarie University Sydney New South Wales Australia; ^6^ Neuroscience Research Australia Randwick New South Wales Australia

**Keywords:** amyotrophic lateral sclerosis, copper ATSM (CuATSM), p62 pathology, riluzole, TDP‐43 pathology

## Abstract

**Aims:**

Although the orally available brain‐penetrant copper compound CuATSM has demonstrated promising effects in SOD1‐linked mouse models, the impact of CuATSM on disease pathology in patients with amyotrophic lateral sclerosis (ALS) remains unknown.

**Methods:**

The present study set out to address this deficit by performing the first pilot comparative analysis of ALS pathology in patients that had been administered CuATSM and riluzole [*N* = 6 cases composed of ALS‐TDP (*n* = 5) and ALS‐SOD1 (*n* = 1)] versus riluzole only [*N* = 6 cases composed of ALS‐TDP (*n* = 4) and ALS‐SOD1 (*n* = 2)].

**Results:**

Our results revealed no significant difference in neuron density or TDP‐43 burden in the motor cortex and spinal cord of patients that had received CuATSM compared with patients that had not. In patients that had received CuATSM, p62‐immunoreactive astrocytes were observed in the motor cortex and reduced Iba1 density was found in the spinal cord. However, no significant difference in measures of astrocytic activity and SOD1 immunoreactivity was found with CuATSM treatment.

**Discussion:**

These findings, in this first postmortem investigation of patients with ALS in CuATSM trials, demonstrate that in contrast to that seen in preclinical models of disease, CuATSM does not significantly alleviate neuronal pathology or astrogliosis in patients with ALS.

## Graphical abstract

This study performs the first postmortem investigation into patients treated with CuATSM during life. Our results demonstrate no significant difference in neuron density or pTDP‐43 burden in patients that had received CuATSM compared to patients that had not.

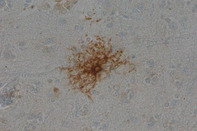

**Key points**

- We perform the first postmortem investigation into patients that had been on the CuATSM trial during life.
- No significant difference in neuron density, TDP burden or measures of astrocytic activity was found in patients that had received CuATSM compared with patients that had not.
- These findings demonstrate that in contrast to that seen in preclinical models of disease, CuATSM does not significantly alleviate neuronal pathology or astrogliosis in patients with ALS.

## INTRODUCTION

Amyotrophic lateral sclerosis (ALS) is a universally fatal and rapidly progressive motor neurodegenerative disease [1, 2]. In rodent models of SOD1‐ALS, the orally available and brain‐penetrant copper compound CuATSM has been found to mitigate symptoms of motor neuron decline and extend survival without adverse effects [3, 4, 5, 6, 7, 8]. However, SOD1‐ALS accounts for only ~2% of all patients with ALS [9, 10], and the translational relevance of CuATSM in treating the ~90% of patients with sporadic disease is less clear. Importantly, whether CuATSM alleviates neuronal pathology in patients with sporadic ALS is not known. The present study set out to address this question in patients that had been administered CuATSM. Given that all patients were also prescribed the glutamatergic modulator riluzole, which is the only approved ALS therapy currently available in Australia, patients with only riluzole treatment were also included in this analysis.

## MATERIALS AND METHODS

### Case selection

Human brain tissue was obtained from the New South Wales Brain Bank, which holds a neuropathologic series collected with informed consent through regional brain donor programs. All cases with pathological confirmation of clinical ALS that had been administered CuATSM (NCT04082832) were selected for this study (*n* = 6) (Table 1). Given that these patients had also been administered riluzole [11], an age‐matched ALS cohort that had been on a similar daily dosage of riluzole was selected (*n* = 6). All cases had previously been staged for topographical progression of TDP‐43 [12, 13] and assessed for genetic mutations in the *C9ORF72*, *TARDBP* and *SOD1* genes. A SOD1 mutation was identified in three cases (p.I114T in CuATSM‐riluzole, p.I114T and p.V149G in riluzole [14]). No other mutations were found. There was no family history of disease in any of the cases without a genetic mutation. This research project was approved by the Human Research Ethics Committees of the Universities of Sydney and New South Wales and complies with the statement on human experimentation issued by the National Health and Medical Research Council of Australia.

**TABLE 1:** Demographic, genetic and drug dosage details in patients.

Case	Group	Age at death (y)	DD (y)	Sex	Postmortem delay (h)	SOD1 mutation	CuATSM Treatment duration (day)	CuATSM taken to death	CuATSM cumulative dose (mg)	Cause of death
1	CuATSM + Ril	<35	5	M	<30	Yes	180	Yes	21,888	ALS
2	CuATSM + Ril	<60	4	M	<35	No	150	Yes	18,612	ALS
3	CuATSM + Ril	<60	2	M	≤5	No	390	Yes	28,080	ALS
4	CuATSM + Ril	<60	2	M	<40	No	210	Yes	21,258	ALS
5	CuATSM + Ril	<55	4	M	≤60	No	270	Yes	27,252	RF
6	CuATSM + Ril	<70	3	M	≤65	No	180	Yes	12,960	ALS
7	Ril	<55	2	F	<25	Yes	0	N/A	0	ALS
8	Ril	<40	5	F	<60	Yes	0	N/A	0	RF
9	Ril	<55	7	M	<75	No	0	N/A	0	ALS
10	Ril	<50	5	M	≤15	No	0	N/A	0	RF
11	Ril	<50	1	M	≤5	No	0	N/A	0	ALS
12	Ril	<60	1	M	≤60	No	0	N/A	0	ALS

Abbreviations: Ril, Riluzole; DD, disease duration; ALS, amyotrophic lateral sclerosis; RF, respiratory failure; M, male; F, female; Y, years; H, hours; mg, milligram; NA, not applicable.

### Immunohistochemistry

Formalin‐fixed, paraffin‐embedded tissue blocks from the motor cortex and spinal cord were sectioned at 10 μm and immunostained with antibodies against phospho‐TDP‐43 (S409/410) (Cosmo Bio Co, TIP‐PTD‐M01, 1:80,000), p62 (BD Biosciences, 610833, mouse, 1:250), GFAP (Agilent, Z033401‐2, rabbit, 1:500), Iba1 (Abcam, ab5076, goat, 1:500) and SOD1 (Merck, 574597, sheep, 1:200) and counterstained with 0.5% cresyl violet as previously described [15]. Immunofluorescence staining was carried out on 10‐μm‐thick sections. Following microwave antigen retrieval (0.01 M citrate buffer, pH 6.0), formaldehyde quenching was carried out using 0.1% sodium borohydride, followed by protein blocking. The sections were then incubated with a primary antibody cocktail consisting of p62 (BD Biosciences, 610833, mouse, 1:250), GFAP (Agilent, Z033401‐2, rabbit, 1:500) and SOD1 (Merck, 574597, sheep, 1:200) antibodies at 4°C overnight. The sections were then visualised with Alexa Fluor 568 donkey anti‐mouse (Thermo Fisher, A10037) and Alexa Fluor 647 donkey anti‐rabbit (Thermo Fisher A‐31573), followed by further blocking with rabbit serum and visualised with AlexaFluor 488 rabbit anti‐sheep (Abcam, 150181). Subsequently, autofluorescence was quenched by Sudan black, and sections were counterstained with DAPI.

### Quantitation of pathologies

Sections were quantified as previously described [15]. In the motor cortex, 2× 500‐μm‐wide strips through the entire cortical thickness from the pial surface to white matter were sampled in each cortical section, and neurons with and without pTDP‐43 and p62 inclusions were counted at 200× magnification using a 10 × 10 eyepiece graticule (500 μm × 500 μm) with standard inclusion (lower and left) and exclusion (upper and right) borders in contiguous, non‐overlapping fields. In the spinal cord, both anterior horns were identified, and neurons with and without pTDP‐43 and p62 inclusions were also counted at 200× magnification using the same 10 × 10 eyepiece graticule. The density of neurons within each region of interest was calculated for each case, and the proportion with pTDP‐43 or p62 inclusions was expressed as a percentage of these. The density of Iba1‐positive microglia was also quantified in this same manner. The areal fraction occupied by GFAP immunopositive astroglia in each region of interest was assessed using a point‐counting method on 200× magnification as previously described [16]. Given that a proportion of astrocytes had obvious p62 immunoreactivity (described in the results), the areal fraction occupied by p62 in each region of interest was also assessed. The proportion of glial cells with p62 or pTDP‐43 was graded on a four‐point scale: 0 = no detectable pathology across the entire section; 1 = mild (some pathology observed in most fields of view at 100× magnification); 2 = moderate; 3 = severe as previously described [17]. Consistent with a recent report [18], diffuse SOD1 immunoreactivity was observed in all ALS cases, and the intensity of these was graded in neurons and glia on a four‐point severity scale of 0–3. Quantitation was performed by two raters blind to case details and treatment group with an inter‐ and intra‐rater variance of <5%.

### Statistics

Statistical analysis was performed using SPSS (Version 25) with a *p*‐value of <0.05 taken as significant. Demographic differences among groups were determined using one‐way ANOVA for age and postmortem delay, and c^2^‐test for gender and presence of SOD1 mutation. Group differences were assessed using multivariate analysis. Correlation analyses were performed with Spearman rank correlation analyses. Consistent with previous reports, SOD1 cases did not demonstrate pTDP‐43 immunoreactivity [19] and, as such, were excluded from analyses of pTDP‐43.

## RESULTS

P62‐positive inclusions were seen in all cases, whereas pathological pTDP‐43 aggregates were only observed in ALS cases without a SOD1 mutation. No pathological brain changes to suggest prolonged hypoxia were observed in the present series. Co‐existing neurodegenerative pathologies were absent.

### Group demographics and daily drug dosage

There were no significant differences in age at death between groups [(mean ± SD) of 55 ± 10 years in the CuATSM group; 50 ± 6 years in the non‐CuATSM group; *p* = 0.5] or disease duration [(mean ± SD) of 3 ± 1 years in the CuATSM group; 3 ± 3 years in the non‐CuATSM group; *p* = 1.0]. No significant difference in postmortem delay [(mean ± SD) of 38 ± 23 h in the CuATSM group; 38 ± 28 h in the non‐CuATSM group; *p* = 0.9], sex (100% male in the CuATSM group; 67% in the non‐CuATSM group; *p* = 0.5) or the presence of SOD1 mutations (17% in the CuATSM group; 33% in the non‐CuATSM group; *p* = 0.5) was identified between groups (*p* > 0.05). All patients in the CuATSM group had received a daily oral dosage of 72 mg. No significant difference in the mean daily riluzole intake was present between groups [(mean ± SD) of 133 ± 52 mg in the CuATSM group; 100 ± 0 mg in the non‐CuATSM group; *p* > 0.1].

### p62‐positive astrocytes in the CuATSM treatment group

**FIGURE 1:**
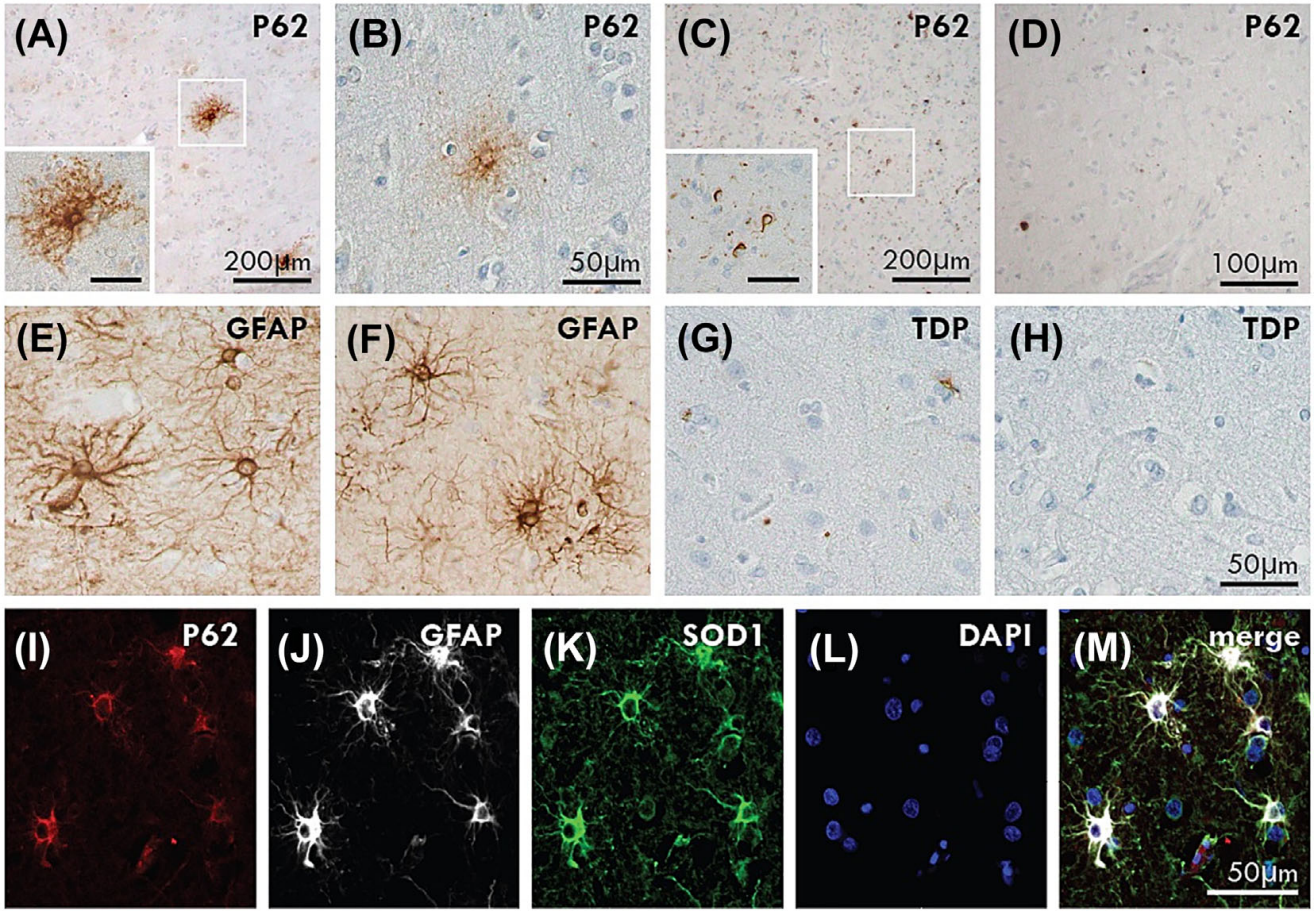
p62‐positive astrocytes in CuATSM treatment group. p62‐immunoreactive astrocytes were observed in the motor cortex of ALS‐TDP (A: Case #3) and ALS‐SOD1 (B: Case #1) cases that had received CuATSM but not in ALS‐TDP (C: Case #11) or ALS‐SOD1 (D: Case #8) cases that had not received CuATSM treatment. The p62‐positive astrocytes were of similar appearance to GFAP‐positive astrocytic morphology (E: Case #3, F: Case #1). pTDP‐positive astrocytes were not seen in ALS‐TDP (G: Case #3) and ALS‐SOD1 (H: Case #1) cases. Immunofluorescent triple labelling demonstrated co‐localisation of p62, GFAP and SOD1 in the p62‐immunopositive astrocytes (I–M). A and C inset scale is 50 μm.

p62‐immunoreactive astrocytes (Figure 1A) were observed in the motor cortex of all ALS cases that had received CuATSM but not in ALS cases that had not (Figure 1D). These astrocytes had typical astrocyte morphology (Figure 1B) and were not immunoreactive for TDP‐43 (Figure 1C). Immunofluorescent triple labelling for p62, GFAP and SOD1 (Figure 1G–K) confirmed these to be p62‐positive astrocytes.

### Group differences

No significant difference in neuron density was found in the motor cortex [(mean ± SD) of 24.8 ± 5.4 in the CuATSM group; 24.5 ± 4.0 in the non‐CuATSM group; *p* > 0.8] or spinal cord [(mean ± SD) of 3.0 ± 1.1 in CuATSM group; 3.4 ± 1.9 in the non‐CuATSM group; *p* > 0.7]. No significant difference in pathological pTDP‐43 burden was observed with CuATSM treatment in either the motor cortex or spinal cord of sporadic cases (Table 2). ALS‐TDP stage was also not significantly different in sporadic cases between treatment groups [(mean ± SD) of 1.2 ± 0.4 in the CuATSM group; 1.7 ± 1.0 in the non‐CuATSM group; *p* > 0.2]. No significant difference in p62 and GFAP burden was found in the CuATSM and non‐CuATSM treatment groups (Table 1). Consistent with a recent report [18], diffuse SOD1 immunoreactivity was identified in all ALS cases irrespective of SOD1 mutation and was similar between cases with compared to without CuATSM treatment. No significant difference in GFAP was observed between treatment groups. However, a significantly lower density of Iba1‐positive microglia was found in the spinal cord of ALS cases that had received CuATSM (*p* = 0.01) (Table 1). No significant difference in Iba1 density was found in the motor cortex.

**TABLE 2:** Mean (±SD) neuronal and glia pathology in patients treated with CuATSM and riluzole or riluzole only.

	ALS‐TDP			SOD1‐ALS		
	CuATSM + Riluzole	Riluzole	*P* value	CuATSM + Riluzole	Riluzole	*P* value
Motor cortex						
Neuron density	25.8 ± 5.7	26.6 ± 2.8	0.8	21.0 ± 0.0	20.2 ± 0.4	N/P
% TDP neurons	0.9 ± 1.5	5.1 ± 4.7	0.1	0.0 ± 0.0	0.0 ± 0.0	N/P
% p62 neurons	2.7 ± 1.4	4.5 ± 4.2	0.5	6.3 ± 0.0	0.7 ± 1.0	N/P
SOD1 neuron burden	2.5 ± 0.4	2.3 ± 0.9	0.6	1.5 ± 0.0	3.0 ± 0.0	N/P
TDP glial burden	1.8 ± 1.2	2.1 ± 1.4	0.7	0.0 ± 0.0	0.0 ± 0.0	N/P
p62 glial burden	2.3 ± 0.9	2.3 ± 0.9	0.9	3.0 ± 0.0	0.5 ± 0.7	N/P
p62 areal fraction	1.0 ± 0.6	0.6 ± 0.5	0.5	0.7 ± 0.0	0.1 ± 0.1	N/P
SOD1 glia burden	2.9 ± 0.3	2.6 ± 0.5	0.4	3.0 ± 0.0	3.0 ± 0.0	N/P
GFAP areal fraction	4.9 ± 1.0	4.4 ± 1.9	0.8	4.9 ± 0.0	3.2 ± 0.3	N/P
Iba1 density	19.0 ± 9.4	17.3 ± 5.3	0.8	24 ± 0.0	14 ± 0.6	N/P
Spinal cord						
Neuron density	3.3 ± 1.0	4.0 ± 2.6	0.6	N/A	3.5 ± 0.7	N/P
% TDP neurons	3.1 ± 6.3	20.0 ± 24.5	0.2	N/A	0.0 ± 0.0	N/P
% p62 neurons	8.1 ± 9.9	29.9 ± 34.8	0.3	N/A	26.2 ± 19.4	N/P
SOD1 neuron burden	2.3 ± 0.9	1.7 ± 0.5	0.3	N/A	1.5 ± 0.0	N/P
TDP glial burden	1.5 ± 1.3	1.3 ± 0.5	0.7	N/A	0.0 ± 0.0	N/P
p62 glial burden	1.5 ± 1.0	1.5 ± 1.0	1.0	N/A	2.0 ± 1.4	N/P
p62 areal fraction	0.4 ± 0.3	0.2 ± 0.2	0.5	N/A	0.4 ± 0.3	N/P
SOD1 glia burden	2.8 ± 0.5	2.4 ± 0.9	0.5	N/A	1.8 ± 1.0	N/P
GFAP areal fraction	2.5 ± 2.5	3.1 ± 2.6	0.8	N/A	4.3 ± 1.1	N/P
Iba1 density	13.3 ± 8.7	36.5 ± 14.5	0.03*	N/A	31.5 ± 10.6	N/P

*Note*: N/A: Tissue from the spinal cord was not available for 1 CuATSM‐riluzole case; N/P: Not performed.

* *p* < 0.05.

### Correlations

The area fraction occupied by GFAP was positively correlated with the proportion of pTDP‐43 neurons in the motor cortex and spinal cord (*r* > 0.58; *p* < 0.05), indicating increased reactive astrocytes with pathological protein deposition in both regions. GFAP was also significantly associated with p62 areal fraction and the proportion of p62‐positive neurons in the spinal cord across all cases (*r* > 0.8, *p* ≤ 0.001) and in ALS‐TDP cases only (*r* > 0.8, *p* ≤ 0.01), indicating increased reactive astrocytes with autophagy. The proportion of pTDP‐43 neurons increased in the motor cortex was found with increasing ALS‐TDP stage (*r* = 0.89, *p* < 0.001). There was no significant correlation between either pTDP‐43 or p62 burden with age at death or disease duration.

## DISCUSSION

CuATSM has been found to meet the copper deficiencies in SOD1 rodent models, mitigating symptoms of motor neuron decline and extending survival [3, 4, 5, 6, 7, 8, 20]. Given that disrupted copper bioavailability has also been found in patients with sporadic ALS, the therapeutic benefits of CuATSM seen in SOD1 models have been proposed to be translatable to patients with the sporadic disease [21]. The present study performs the first postmortem investigation into patients that had been treated with CuATSM during life. Our results revealed no significant difference in neuron density or TDP burden in patients that had received CuATSM compared with patients that had not. In contrast to that seen in preclinical models [3, 4, 5, 6, 8, 22, 23], SOD1 immunoreactivity and GFAP expression were similar in CuATSM and non‐CuATSM treatment groups. However, patients treated with CuATSM demonstrated p62‐immunoreactive astrocytes in the motor cortex and reduced Iba1 density in the spinal cord.

P62 is an autophagy substrate that plays a critical role in aggregate degeneration [24] and was observed here in motor cortical astrocytes of CuATSM‐treated patients only, where it filled the astrocyte cytoplasm in a similar way to GFAP (Figure 1). Although p62‐immunopositive astrocytes have not been described in postmortem tissue of patients with ALS, elevated p62 levels have been identified in reprogrammed skin‐derived astrocytes from patients with sporadic, *SOD1* and *C9ORF72* ALS [23, 25]. Importantly, no relationship between astroglial sequestosome activity with CuATSM treatment was found in patient cell lines [23], suggesting that the p62‐astrocytes observed here do not reflect a therapeutic response to CuATSM. Further to this, in contrast to the reduced astrocytic activity seen in SOD1 rodent models [3, 4, 5, 6, 7, 8], no significant change in measures of GFAP was found with CuATSM here. Instead, lower levels of activated microglia were observed in the spinal cord of patients with CuATSM treatment. Interestingly, a negative correlation between disease duration and activated microglia has been reported in the spinal cord of ALS cases [26], but this was not observed in the present cohort. As is the case for most quantitative pathological studies, the main methodological issue warranting consideration is the relatively small sample sizes, and future replication of these results in a larger sample is needed. Nevertheless, the assessment of tissue from six patients that had been on the CuATSM trial is significant, and the striking and consistent findings provide strong support to suggest our results are representative.

In summary, this first postmortem analysis of patients treated with CuATSM demonstrates p62‐immunoreactive astrocytes in the motor cortex and lower Iba1‐positive microglia in the spinal cord. Importantly, however, no significant difference in neuronal density and the pathological burden was found with CuATSM treatment, indicating no significant pathological benefit associated with this drug in this cohort. Future studies will be needed to determine whether specific subsets of patients may benefit from this drug.

## AUTHOR CONTRIBUTIONS

Data acquisition and analysis: Yue Yang, Dominic Rowe, Heather McCann, Claire E Shepherd, Jillian J Kril, Matthew C Kiernan; study conception, design, analysis and interpretation: Rachel H Tan and Glenda M Halliday; writing and revision of the manuscript: Rachel H Tan. All authors have read and approved the final manuscript.

## CONFLICT OF INTEREST STATEMENT

The authors declare that they have no competing interests.

## DISCLOSURE STATEMENT

The authors have no conflict of interest to report. The Editors of Neuropathology and Applied Neurobiology are committed to peer‐review integrity and upholding the highest standards of review. As such, this article was peer‐reviewed by independent expert referees and the authors (GMH) had no role in either the editorial decision or the handling of the paper.

## ETHICS APPROVAL

This research project was approved by the Human Research Ethics Committees of the Universities of Sydney and New South Wales and complies with the statement on human experimentation issued by the National Health and Medical Research Council of Australia. Tissues were selected from a neuropathological series collected by the NSW Brain Banks through regional brain donor programs in Sydney, Australia. The brain donor programs hold approval from the Human Research Ethics Committees of the South Eastern Sydney Area Health Services and comply with the statement on human experimentation issued by the National Health and Medical Research Council of Australia.

## Data Availability

The data that support the findings of this study are available on request from the corresponding author. The data are not publicly available due to privacy or ethical restrictions.
